# In-Depth Characterization of L1CAM^+^ Extracellular Vesicles as Potential Biomarkers for Anti-CD20 Therapy Response in Relapsing–Remitting Multiple Sclerosis

**DOI:** 10.3390/ijms26157213

**Published:** 2025-07-25

**Authors:** Shamundeeswari Anandan, Karina Maciak, Regina Breinbauer, Laura Otero-Ortega, Giancarlo Feliciello, Nataša Stojanović Gužvić, Oivind Torkildsen, Kjell-Morten Myhr

**Affiliations:** 1 Department of Clinical Medicine, University of Bergen, 5021 Bergen, Norway; oivind.fredvik.grytten.torkildsen@helse-bergen.no (O.T.); kjell-morten.myhr@helse-bergen.no (K.-M.M.); 2 Neuro-SysMed, Department of Neurology, Haukeland University Hospital, 5021 Bergen, Norway; 3 Department of General Biochemistry, Faculty of Biology and Environmental Protection, University of Lodz, 90-136 Lodz, Poland; karina.maciak@edu.uni.lodz.pl; 4 Faculty of Medicine, Friedrich-Alexander-University Erlangen-Nuremberg (FAU), 91054 Erlangen, Germany; regina.breinbauer@fau.de; 5 Neurology and Cerebrovascular Diseases Group, Neurology Department, Neurosciences Area, La Paz Hospital Institute for Health Research, 28046 Madrid, Spain; oteroortega.l@gmail.com; 6 Fraunhofer Institute for Toxicology and Experimental Medicine (ITEM-R), Personalized Tumor Therapy, 93053 Regensburg, Germany; giancarlo.feliciello@item.fraunhofer.de (G.F.); natasa.stojanovic@item.fraunhofer.de (N.S.G.)

**Keywords:** multiple sclerosis (MS), brain-derived blood exosomes, anti-CD20 therapy, rituximab, treatment-response biomarkers, characterization of L1CAM^+^ EVs

## Abstract

The effective suppression of inflammation using disease-modifying therapies is essential in the treatment of multiple sclerosis (MS). Anti-CD20 monoclonal antibodies are commonly used long-term as maintenance therapies, largely due to the lack of reliable biomarkers to guide dosing and evaluate treatment response. However, prolonged use increases the risk of infections and other immune-mediated side effects. The unique ability of brain-derived blood extracellular vesicles (EVs) to cross the blood–brain barrier and reflect the central nervous system (CNS) immune status has sparked interest in their potential as biomarkers. This study aimed to assess whether blood-derived L1CAM^+^ EVs could serve as biomarkers of treatment response to rituximab (RTX) in patients with relapsing-remitting MS (RRMS). Serum samples (*n* = 25) from the baseline (month 0) and after 6 months were analyzed from the RTX arm of the ongoing randomized clinical trial OVERLORD-MS (comparing anti-CD20 therapies in RRMS patients) and were compared with serum samples from healthy controls (*n* = 15). Baseline cerebrospinal fluid (CSF) samples from the same study cohort were also included. EVs from both serum and CSF samples were characterized, considering morphology, size, and concentration, using transmission electron microscopy (TEM) and nanoparticle tracking analysis (NTA). The immunophenotyping of EV surface receptors was performed using flow cytometry with the MACSPlex exosome kit, while label-free quantitative proteomics of EV protein cargo was conducted using a proximity extension assay (PEA). TEM confirmed the presence of EVs with the expected round morphology with a diameter of 50–150 nm. NTA showed significantly higher concentrations of L1CAM^+^ EVs (*p* < 0.0001) in serum total EVs and EBNA1^+^ EVs (*p* < 0.01) in serum L1CAM^+^ EVs at baseline (untreated) compared to in healthy controls. After six months of RTX therapy, there was a significant reduction in L1CAM^+^ EV concentration (*p* < 0.0001) and the downregulation of TNFRSF13B (*p* = 0.0004; FC = −0.49) in serum total EVs. Additionally, non-significant changes were observed in CD79B and CCL2 levels in serum L1CAM^+^ EVs at baseline compared to in controls and after six months of RTX therapy. In conclusion, L1CAM^+^ EVs in serum showed distinct immunological profiles before and after rituximab treatment, underscoring their potential as dynamic biomarkers for individualized anti-CD20 therapy in MS.

## 1. Introduction

Almost 3 million people worldwide are affected by multiple sclerosis (MS), an inflammatory and degenerative disease of the central nervous system (CNS) [1]. Initially, most patients (85–90%) experience a relapsing-remitting course (RRMS) marked by episodic inflammation and, if not effectively treated, followed by a secondary progressive (SPMS) phase, associated with gradual increasing disability. A primary progressive course (PPMS) is seen in approximately 10–15% of cases [2].

Strong epidemiological evidence suggests that Epstein–Barr Virus (EBV) is a prerequisite for developing MS, although the exact underlying pathogenic mechanisms remain unclear [3,4]. EBV is transmitted through saliva and, upon infecting B-cells, enters a state of “deep” latency, where it transfers its DNA to the B-cell nucleus, with the possibility to still be replicated along with the cellular DNA and be reactivated following B-cell activation [3,5].

The suppression of inflammatory activity is the cornerstone of MS treatment with disease-modifying therapies [6,7]. Evolving clinical experience from anti-CD20 therapies has shown their high efficacy in relapsing forms of the disease. Anti-CD20 therapy is also the first treatment approach proven to modify disability worsening in PPMS [8]. It reduces inflammatory activity, usually shown by the almost complete prevention of new clinical relapses and new brain magnetic resonance imaging (MRI) lesions in treated patients [9]. Anti-CD20 therapies in current practice internationally include rituximab, ocrelizumab, ofatumumab, and most recently ublituximab [8,10].

Anti-CD20 therapies are typically initiated with an induction dose, followed by regular fixed maintenance dosing at regular intervals. While convenient, these fixed regimens may result in overtreatment, as B-cell reconstitution often occurs long after B-cell depletion, with considerable interindividual variability. B-cell counts frequently remain suppressed at the time of scheduled redosing, with immune reconstitution ranging from 27 to 125 weeks post-depletion (median: 72 weeks) [11]. Notably, approximately 6% of total circulating T-cells express CD20, and transient drops in T-cell numbers occur for 3–6 months following anti-CD20 treatment, possibly related to the depletion of CD20-positive T-cells [11,12,13,14].

Together, these data suggest that a reprogramming of immunity from an activated to a resting state may occur following therapy and could account for the high efficacy of treatment [8].

In MS treatment, CD19 (B-cell marker) is frequently used as a surrogate measure to quantify B-cells during treatment. A recent study showed that the use of memory B-cell counts as treatment monitoring biomarkers in rituximab reinfusion protocols can reduce the mean number of infusions with a persistent reduction in disease activity [15]. However, existing data are insufficient for establishing a therapeutic cut-off of B-cells in RRMS [16]; as anti-CD20 therapies deplete pre-B-cells to late plasma blasts (including CD20^+^ T-cells), using only CD19 counts as biomarkers might therefore be oversimplistic, compelling the need for more robust biomarkers [16].

Extracellular vesicles (EVs) are membrane-bound particles, released by virtually all cell types. They can exert their action locally or migrate to distant locations via biological fluids (urine, cerebrospinal fluid (CSF), peripheral blood, saliva, breast milk, tears) [17]. EVs process a sophisticated cargo-sorting mechanism, carrying lipids, proteins, nucleic acids, and specific membrane proteins that largely reflect their cell of origin. These characteristics, combined with their ability to cross the blood–brain barrier (BBB) and increased stability, highlight brain-derived blood EVs as promising biomarkers for CNS diseases, including MS [18,19,20,21].

Recent research on brain-derived blood EVs in MS has particularly focused on L1 cell adhesion molecule (L1CAM), in addition to other markers like glutamate aspartate transporter (GLAST) and myelin oligodendrocyte glycoprotein (MOG). These studies primarily investigate the potential of these biomarkers related to disease activity and progression [3,22,23,24,25,26,27]. Despite this cumulative evidence, the utility of L1CAM as a marker of brain/neuron-derived EVs is still under debate as it is also expressed at comparable RNA levels by Schwann cells of the peripheral nervous system, skin epithelial cells, and kidney tubule epithelia and may occur as free L1CAM peptides [28,29,30,31,32]. This study is the first to comprehensively characterize L1CAM^+^ EVs, assessing size, concentration, morphology, protein cargo, and surface immunophenotype, in both serum and CSF, while also evaluating their utility as dynamic biomarkers for anti-CD20 treatment response in RRMS.

We hypothesize that L1CAM^+^ EVs reflect central immune activity and could serve as serum biomarkers to monitor treatment response to anti-CD20 therapy.

## 2. Results

### 2.1. Patient Characteristics and EV Characterization: Size, Morphology, and Tetraspanin Profiling

**Figure 1:**
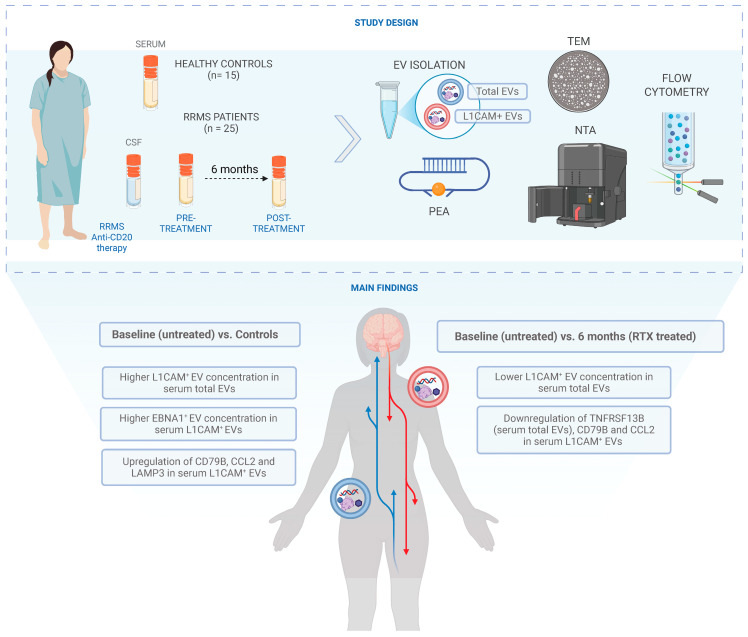
Schematic overview of the study design, analysis set-up, and main findings: Significantly higher concentrations of L1CAM^+^ EVs (*p* < 0.0001) in serum total EVs and EBNA1^+^ EVs (*p* < 0.01) in serum L1CAM^+^ EVs at baseline (untreated) compared to in healthy controls were observed. After six months of RTX therapy, there was a significant reduction in L1CAM^+^ EV concentration (*p* < 0.0001) and the downregulation of TNFRSF13B (*p* = 0.0004; FC = −0.49) in serum total EVs.

Total EVs and L1CAM-enriched EVs (L1CAM^+^ EVs) were analyzed in serum samples from 25 newly diagnosed, treatment-naïve RRMS patients at baseline (month 0) and after six months (month 6) of rituximab therapy, who all showed no signs of new disease activity. In addition, diagnostic CSF samples from the same cohort (month 0) and serum control samples from 15 healthy individuals were included (see Figure 1).

**Figure 2:**
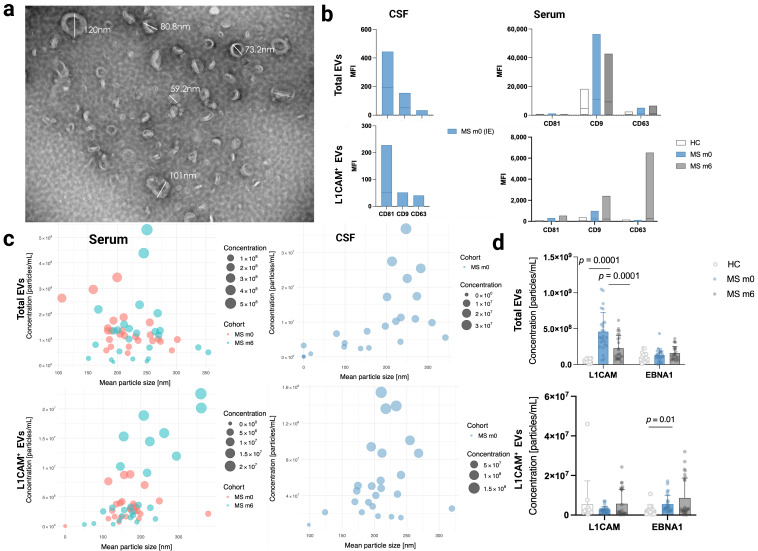
Characteristics of serum and CSF EVs: (**a**) Morphology detected with TEM imaging (scale bar: 200 nm) and (**b**) tetraspanin (CD81, CD9, and CD63) (bead population numbers: 65, 53, and 56, respectively—flow cytometry analysis) expression profile of MS patients (month 0—m0 and month 6—m6) and healthy controls, with total EVs and L1CAM^+^ EVs expressed in mean fluorescence intensities (MFIs) as floating bar plots with min. and max. ranges. NTA: (**c**). Bubble plots show the relationship between mean EV particle size and the concentration of total EVs and L1CAM^+^ EVs. Each point represents an individual sample, with bubble size proportional to EV concentration. (**d**). L1CAM and EBNA concentration in serum total EVs and L1CAM^+^ EVs. Significantly higher concentrations of L1CAM^+^ EVs (*p* < 0.0001) in serum total EVs and EBNA1^+^ EVs (*p* < 0.01) in serum L1CAM^+^ EVs at baseline (untreated) compared to in healthy controls were observed. After six months of RTX therapy, there was a significant reduction in L1CAM^+^ EV concentration (*p* < 0.0001). Statistical significance between HC and m0 was assessed using the U Mann–Whitney test. Statistical significance between m0 and m6 was determined using the Wilcoxon signed-rank test.

The cohort predominately consisted of women (80%), with a mean age of 39.8 (SD = 10.36) years and stable disease. TEM imaging revealed the expected double-membraned, rounded morphology of EVs with a diameter range of 50–150 nm (see Figure 2a and Figure S1c: pilot experiment results; *n* = 5). The profiling of tetraspanins (CD9, CD63, CD81), which are common EV transmembrane markers, showed the highest expression of CD9 in serum total EVs, CD63 in serum L1CAM^+^ EVs, and CD81 in both total and L1CAM^+^ CSF EVs (see Figure 2b and Figure S1b).

### 2.2. Significant Changes in L1CAM^+^ EV Concentrations Before and After Rituximab-CD20 Treatment in Serum Total EVs

**Figure 3:**
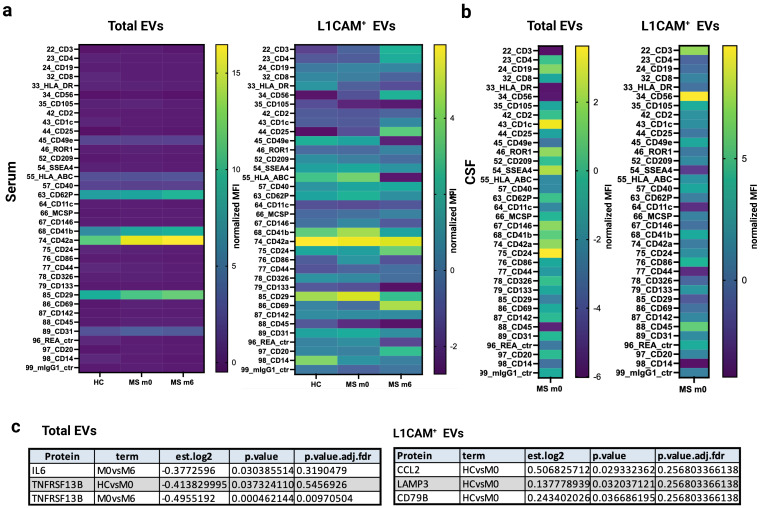
(**a**,**b**). Surface immune profiling of total and L1CAM^+^ EVs shown as heatmaps (EV markers’ geometric mean fluorescence intensity (MFI) was normalized to the mean MFI for specific EV markers (CD9, CD63, and CD81), obtaining normalized MFI values) from CSF and serum samples comparing MS patients (m0, m6) and healthy controls. Statistical significance between HC and m0 was assessed using the U Mann–Whitney test and Wilcoxon signed-rank test (m0 and m6) with no significant findings. (**c**). Linear regression models adjusted for sex and age were used to estimate differences over time (MS = m0 and m6) and between sample groups (HC and MS) for the 21-flex panel proteins. Differentially expressed proteins with significant changes in both serum total and L1CAM^+^ EVs are presented. After six months of RTX therapy, there was a significant downregulation of TNFRSF13B (*p* = 0.0004; FC = −0.49) in serum total EVs. The models were adjusted for the covariates ‘age’ and ‘sex’, and inferential tests were two-tailed with a nominal alpha level of 0.05. Raw *p*-values were adjusted for multiple testing by controlling the false discovery rate with the Benjamini and Hochberg method, and the critical value (q-value) was set to ≤0.01.

NTA revealed a significantly increased concentration of L1CAM^+^ EVs (*p* < 0.0001) in serum total EVs at baseline (untreated) compared to in healthy controls (see Figure 2d). The expression of 21 immune and inflammation-related proteins (see Supplementary Table S1—PEA analysis) were assessed in serum L1CAM^+^ EVs; CD79B (*p* = 0.03; fold change (FC) = 0.24) and CCL2 (*p* = 0.02; fold change (FC) = 0.50), demonstrated differential expression at baseline prior to rituximab therapy compared to controls, although these differences were non-significant after adjustments (Figure 3c and Supplementary Table S2).

Interestingly, matched CSF total EVs also showed elevated concentrations of CCL2 (FC = 9.35) and TREM2 (FC = 10.71) at baseline (see Supplementary Table S4). The immunophenotyping of 37 surface markers though flow cytometric analysis indicated the elevated (ns) expression of CD41b, CD42a, and CD29 in serum L1CAM^+^ EVs at baseline compared to healthy serum controls. Additionally, the increased expression of CD1c and CD24 was observed in CSF total EVs at baseline (see Figure 3a,b and Supplementary Table S6).

The concentration of L1CAM^+^ EVs in serum total EVs significantly decreased (*p* < 0.0001) after rituximab treatment at month 6 compared to baseline, as indicated by NTA, (see Figure 2d). The serum total EV cargo exhibited the significant downregulation of TNFRSF13B (*p* = 0.0004; FC = −0.49) and non-significant downregulation of CD79B (ns; FC = −0.02), based on PEA analysis (see Figure 3 and Supplementary Table S3).

Additionally, the serum L1CAM^+^ EV cargo showed a non-significant downregulation of CCL2 (ns; FC = −0.14) and the upregulation of TNF (ns; FC = 0.11), IL-4 (ns; FC = 1.70), and VSNL1 (ns; FC = 0.09) at month 6 after rituximab therapy compared to at baseline (see Figure 3c and Supplementary Tables S2 and S3). Matched CSF L1CAM^+^ EVs also demonstrated increased concentrations of IL-4 (FC = 9.35) and TREM2 (FC = 3.2) at baseline (see Supplementary Table S5).

Surface immune receptor profiling revealed the elevated (ns) expression of CD3 and CD56 in CSF L1CAM^+^ EVs at baseline, as well as CD42a, CD24, CD69, and CD25 in serum L1CAM^+^ EVs at month 6 compared to at baseline (Figure 3 and Supplementary Table S6).

### 2.3. Significantly Elevated EBNA1^+^ EV Concentration in Serum L1CAM^+^ EVs at Baseline Before Rituximab Treatment Compared to HC

EBV is considered as a strong prerequisite for developing MS and Epstein–Barr nuclear antigen 1 (EBNA1), which is known to be expressed in all known latent types of EBV infection. The NTA revealed a significantly increased concentration of EBNA1^+^ EVs (*p* < 0.01) in serum L1CAM^+^ EVs at baseline compared to in controls (see Figure 2d and Figure S1). Additionally, an upregulated expression of LAMP3 (*p* = 0.03; FC = 0.13) was observed in L1CAM^+^ EVs at baseline compared to in controls, although this was non-significant after adjustment (PEA analysis—see Figure 3c and Supplementary Table S2).

The upregulation of LAMP3 (ns; FC = 0.11) was also noted in serum total EVs at month 6 compared to at baseline (Supplementary Table S3). The immunophenotyping of the surface receptors in serum total EVs showed the elevated (ns) expression of CD29, CD42a, CD41b, and CD62p at month 6 compared to at baseline (see Figure 3a).

## 3. Discussion

In this study, we assessed whether L1CAM^+^ blood-derived EVs could serve as biomarkers for guiding personalized rituximab (anti-CD20) therapy in RRMS. As demonstrated in Figure 2 and Figure 3, L1CAM, EBNA1, and selected immune-related proteins (CD79B, CCL2, TNFRSF13B) were dynamically regulated in response to rituximab therapy, particularly in L1CAM^+^ EVs.

During the course of MS, immune cells release EVs, which provide important information regarding ongoing pathological processes [33,34,35,36]. EVs have been studied in the context of MS for their roles in inflammation and T-cell activation. However, previous research has primarily focused on total circulating EVs, without discriminating between specific cell subpopulations [20,37].

Currently available methods to examine brain processes in MS include clinical and MRI assessments, as well as CSF, which involve the invasive procedure of lumbar puncture and serum markers of axonal damage or astrocyte activation [38]. Recent advances in serum EV analysis have expanded the possibilities to study subpopulations of EVs from specific cellular origins, creating new opportunities to assess processes in the brain and the involvement of the immune system in the disease [34,39]. Research on brain-derived blood EV research in MS, particularly focusing on L1CAM, alongside other subpopulations such as GLAST and MOG, has gained attention recently, primarily examining the potential of these EVs as biomarkers in relation to disease status and treatment response [3,22,23,24,25,26,27].

B and T lymphocytes play crucial roles in the pathogenesis of MS. Recent studies have shown that increased levels of CCR5 in Th1-derived EVs and CCR3 in Th2-derived EVs are strong indicators of disease activity, particularly in the presence of gadolinium-enhancing lesions in the brain and spinal cord [39]. Additionally, increased levels of CD19^+^ B-cell-derived EVs have been observed in patients during clinical relapses compared to periods of remission [39].

Similarly, our findings indicate a significant increase (*p* < 0.0001) in the concentration of L1CAM^+^ EVs within total EVs and the upregulation of CD79B (*p* = 0.03; FC = 0.24; ns after adjustment), a protein subunit of the B-cell receptor, as well as CCL2 (*p* = 0.02; FC = 0.50; ns after adjustment), specifically at baseline prior to rituximab therapy compared to in controls in both serum total and L1CAM^+^ EVs. Furthermore, we noted the downregulation of these proteins in L1CAM^+^ EVs along with TNFRSF13B (*p* = 0.0004; FC = −0.49)—a gene that produces TACI, a B-cell-specific member of the TNF receptor superfamily. A significant decrease (*p* < 0.0001) in L1CAM^+^ EV concentration in total EVs was also observed at month 6 compared to at baseline in total serum EVs.

Another study indicated that higher levels of T-cell-derived EVs and smaller sizes of neuron-derived EVs were associated with clinical relapses [22]. CCR2 is the major receptor for CCL2 and functions as a potent chemoattractant for monocytes and T-cells. Chemokines and their receptors are vital for the bidirectional trafficking of leucocytes across the BBB. Several studies have explored the significance of CCL2 and CCR2 in MS, revealing that CCL2 levels are consistently low in the CSF, despite being abundantly expressed within the CNS lesions. These studies suggest that CCL2 is consumed by migrating inflammatory cells, which downregulate CCR2, as they cross the BBB [40].

In line with our findings, CSF total EVs showed the upregulation of CCL2 (FC = 9.35) at baseline prior to rituximab therapy. Interestingly serum L1CAM^+^ (brain-derived) EVs exhibited higher CCL2 expression (*p* = 0.02; ns after adjustment; FC = 0.50) compared to serum total EVs (ns; FC = 0.28) at baseline. Additionally, Iglesias et al. investigated B-cell-derived EVs from the blood and CSF for their myelin antibody content from 136 MS patients, 23 white matter brain lesion controls, and 39 healthy controls. They found autoreactive myelin antibodies in EVs released by peripheral B-cells but not by populations of B-cells resident in CSF [23].

Furthermore, another study examined antibody titers against nuclear (anti-EBNA1) and capsid (anti-VCA) EBV antigens in EVs and in plasma, also evaluating the content of myelin antibodies in EVs [3]. Patients with active disease showed higher levels of anti-EBNA1 in EVs than patients with inactive disease [3]. Correspondingly, our findings revealed increased EBNA1 (*p* < 0.01) concentrations and the upregulation of LAMP3 (*p* = 0.03; ns after adjustment; FC = 0.13) in serum L1CAM^+^ EVs at baseline, indicating higher disease activity in newly diagnosed patients prior to initiation of rituximab therapy compared to HC.

This study has several strengths, including the availability of paired CSF and serum samples at baseline, in addition to a well-characterized cohort of newly diagnosed, treatment-naïve patients who all received the same disease-modifying therapy, rituximab. However, the study also has limitations, most notably its limited sample size (*n* = 25), the absence of long-term clinical outcomes beyond six months, and a lack of replication in an independent cohort with a critical comparison including other disease-modifying therapies or patients with active disease activity. Additionally, as mentioned before, while L1CAM is commonly used to enrich CNS-derived EVs, its expression can also be detected in other peripheral tissues; hence, further recommended validations of neuronal origin with additional markers such as GAP43, β-III-tubulin and VAMP2 based on new published findings should be rigorously followed up [29,32]. Nevertheless, our findings suggest that L1CAM^+^ serum EVs may serve as promising biomarker for treatment response biomarkers in RRMS. Validation in larger, longitudinal cohorts with clinical outcome data is essential. If validated, L1CAM^+^ EVs could facilitate more flexible and individualized dosing schedules for anti-CD20 therapy, potentially reducing side effects associated with unnecessary immune suppression.

## 4. Materials and Methods

### 4.1. Patient Cohorts and Control Samples

Diagnostic CSF (*n* = 25; month 0) and serum samples (*n* = 25) from baseline (month 0) and after six months of treatment were obtained from RRMS patients receiving rituximab as a part of an ongoing randomized clinical trial (OVERLORD-MS: https://clinicaltrials.gov/ct2/show/NCT04578639 (accessed on 1 September 2024)) at Haukeland University Hospital, Bergen, Norway. Clinical evaluations included a history of relapses and disability assessment using the Expanded Disability Status Scale (EDSS). The OVERLORD-MS study was approved by the Regional Committee for Medical and Health Research Ethics, Western Norway—REC West ID: 66391. All participating patients provided informed consent for treatment response biomarker research.

Serum control samples (*n* = 15) were collected from heathy volunteers with informed consent approved under REC West ID: 74985. Clinical samples from all cohorts were collected and handled in accordance with relevant guidelines and regulations. Pilot experiments for the initial set-up of various analyses (Supplementary Figures S1 and S2) also included serum samples from the OVERLORD-MS study.

In briefly, 2 × 6–8 mL blood was collected without anticoagulant. Following centrifugation at 1400× *g* for 12 minutes (min) at room temperature, the blood clotted, and serum was frozen at −80 °C without any additives. CSF samples, upon collection, were centrifuged first at 300× *g* for 15 min at 4 °C, followed by a second centrifugation at 680× *g* for 10 min at 4 °C, before being frozen at −80 °C without any additives.

### 4.2. Isolation of EVs from CSF and Serum Samples

Frozen serum and CSF samples (−80 °C) were uniformly thawed using a thermomixer (Eppendorf, Hamburg, Germany) at 10 °C for 20 min. Total EVs were isolated according to the manufacturer’s instructions. The total exosome isolation kit for other body fluid kits was used for CSF samples, while the total exosomes isolation serum kit (Invitrogen—life technologies, Thermo Fischer Scientific, Carlsbad, CA, USA) was utilized for serum samples.

From the total EVs, L1CAM^+^ EVs were further isolated using the exosome–streptavidin isolation/detection kit (Invitrogen—life technologies, Thermo Fischer Scientific, Carlsbad, CA, USA). The initial step involved coupling of dynabeads^®^, magnetic beads with a biotinylated antibody, CD171 (CD171/L1CAM monoclonal antibody clone eBio5G3 (5G3), Biotin, eBioscience, Thermo Fischer Scientific, Carlsbad, CA, USA), following the manufacturer’s protocol. These total and immuno-purified L1CAM^+^ EVs were subsequently characterized as explained below (Section 4.3).

### 4.3. Characterization of EVs

#### 4.3.1. Transmission Electron Microscopy (TEM) Imaging

A droplet of intact EVs (10 μL resuspended in water; total serum EVs required an additional dilution of 1:30) was placed on a glow-discharged 200 mesh formvar carbon coated copper grid to be absorbed for 1 min. Excess sample was then removed using blotting paper, and the grids were washed once with milli-Q water before being stained for 30 sec in 2% uranyl acetate. The grids were allowed to dry for 30 min before imaging was conducted using a Hitachi HT7800 transmission electron microscope (Minato-ku, Tokyo, Japan).

#### 4.3.2. Nanoparticle Tracking (NTA) Analysis

EV size, concentration, L1CAM content, and EBNA1 content were determined using nanoparticle tracking analysis (NTA) with the NanoSight NS500 nanoparticle analyzer (Malvern Instruments, Malvern, Worcestershire, UK). Frozen EV samples (both total and L1CAM^+^ EVs) were thawed at 4 °C and immediately prepared for staining. For each sample, 5 μL of EV suspension was incubated on ice for at least 30 min with the following reagents: CellMask™ Green dye (1:53; Thermo Fisher Scientific, Waltham, MA, USA) to assess the total quantity of EVs in the sample; L1CAM antibody conjugated to Alexa Fluor^®^ (1:2; Santa Cruz Biotechnology, Dallas, TX, USA) to identify L1CAM content in EVs; and EBNA1 antibody conjugated to phycoerythrin (PE) (1:36; Bio-Techne R&D Systems, Minneapolis, MN, USA) to quantify EBNA1 content in EVs. All staining procedures were performed on ice to preserve vesicle integrity.

For L1CAM^+^ EVs, an aliquot of the EV suspension was diluted 1:50 in sterile PBS, while for total EV quantification, samples were diluted to a final volume of 1:500 in sterile PBS. Measurements were performed using laser excitation of 405 nm for EBNA-1 detection using the PE-conjugated antibody, 535 nm for CellMask™ Green, and 488 nm for L1CAM detection using the Alexa Fluor^®^-conjugated antibody. For each patient sample, three 60 s videos were recorded, capturing 30 frames per position at a detection threshold of 5. The mean particle diameter and concentration were calculated for each run, and the average of the triplicate measurements was used for further analysis.

For pilot experiments (Supplementary Figure S1), frozen EV samples were thawed at 4 °C. A total of 20 µL of prediluted total EVs (1:100) were stained for a minimum of 30 min with 1 µL CellMask™ Green-CMG dye Plasma Membrane Stain (Thermo Fisher Scientific, pre-diluted 1:20). Total EVs were additionally stained with 1 µL of 1:50 pre-diluted EBV EBNA-1 antibody (clone 1EB12) conjugated to Phycoerythrin-PE dye (Santa Cruz Biotechnology, Inc., Dallas, TX, USA) on ice.

The stained samples were further diluted to a final volume of 500 µL in phosphate-buffered saline (PBS, Gibco, Waltham, MA, USA), resulting in final dilution factors of 1:2500 for total EVs and 1:25 for L1CAM^+^ EVs. These samples were then analyzed using a ZetaView^®^ (Particle Metrix, Ammersee, Germany). The manufacturer’s default software settings were employed for EV analysis. For each measurement, three cycles were performed by scanning 11 cell positions and capturing 30 frames per position. For measuring EBNA1- or CMG-stained particles, a 550/25 nm long-pass (LP) fluorescence filter with a sensitivity of 96 and a trace length of 7 (for PE-conjugated antibody) or 10 (for CMG) was used. Data analysis was performed with built-in ZetaView Software version 8.05.14 SP7.

#### 4.3.3. Flow Cytometry

The MACSPlex Human Exosome Kit (Miltenyi, Bergisch Gladbach, Germany) was utilized to examine the surface immune profile of both the total and immuno-purified L1CAM^+^ EVs following the staining procedure as per the manufacturer’s instructions (short protocol for the assay using 1.5 mL tubes). The analyses were performed using a conventional BD Fortessa flow cytometer (BD Biosciences, Franklin Lakes, NJ, USA) with the standard set-up recommended from the kit (Supplementary Table S6).

Briefly, EVs were incubated with antibody-coated MACSPlex exosome capture beads and then labeled with the MACSPlex exosome detection reagents, which included CD9, CD63, and CD81. These complexes were subsequently analyzed based on the fluorescence characteristics of both the MACSPlex Exosome Capture Bead and the detection reagents.

#### 4.3.4. Proximity Extension Assay (PEA)

CSF and serum samples were sent in one batch to Olink Proteomics (Bevital AS, Bergen, Norway) for the quantification of 21 immune and inflammation-related proteins included in the Olink Flex panel (see Supplementary Table S1) using proximity extension assay (PEA).

In brief, the PEA utilizes single-stranded DNA (ssDNA) oligonucleotides covalently attached to pairs of protein-specific antibodies. When both antibodies bind in close proximity to their target protein, their ssDNA oligos hybridize to form a double-stranded DNA (dsDNA) sequence. The Olink Flex procedure requires a minimal sample volume of 1 μL and no replicates and consists of three core steps: incubation (antibody binding), extension/amplification (DNA reporter generation), and detection (qPCR quantification using the Olink Signature Q100 instrument, Waltham, MA, USA).

Internal controls, incubation, extension, and detection controls monitor assay performance in each step, with the extension control normalizing technical variation between samples. NPX (Normalized Protein eXpression), a log2-scale relative quantification unit, is calculated by adjusting cycle threshold (Ct) values using the extension control, a pre-determined bridging factor, and triplicate calibrator. Absolute quantification in pg/mL is achieved by fitting NPX values to protein-specific 4PL model standard curves derived during validation. For details, see the service provider’s homepage (https://olink.com/products/olink-flex (accessed on 1 December 2024)).

### 4.4. Data Analysis

Following the initial gating strategy of the flow data (as per MACSPlex Human Exosome Kit—data analysis protocol), using FlowJo™ v10 software (BD Biosciences, Franklin Lakes, NJ, USA), an equal number of events across both timepoints (m0 and m6) and controls within each sample subpopulation (serum L1CAM^+^ EVs = 15,290 events; serum total EVs = 6028 events; CSF L1CAM^+^ EVs = 19,571 events; CSF total EVs = 10,680 events) was used for further analysis. All downstream analyses were based on normalized geometric mean fluorescence intensity (nMFI) values. In short, a blank control composed of only MACSPlex buffer, incubated with beads and MACSPlex exosome detection reagents (CD9, CD63, and CD81), was used to measure the background signal. Each EV marker’s geometric mean fluorescence intensity (MFI) was normalized to the mean MFI for specific EV markers (CD9, CD63, and CD81) obtaining the normalized MFI (Supplementary Figure S3).

ZetaView Software version 8.05.14 SP7 was utilized for both acquisition and analysis. In PEA analysis, between-group differences (MS vs. HC) in protein levels at baseline (m0) and follow-up (m6) were analyzed by linear regression (generalized least squares) adjusting for heterogeneity in variance between groups using the gls function in the R package nlme v3.1-159. Change scores from baseline to follow-up in the group of MS patients were analyzed by the same linear model that included an unstructured covariance matrix and a variance function structure allowing for different variance per stratum of ‘time’. The models were adjusted for the covariates ‘age’ and ‘sex’, and inferential tests were two-tailed with a nominal alpha level of 0.05. Raw *p*-values were adjusted for multiple testing by controlling the false discovery rate with the Benjamini and Hochberg method, and the critical value (q-value) was set to ≤0.01. In general, statistical analysis was performed using Microsoft Excel (Redmond, WA, USA) and GraphPad Prism 10 (San Diego, CA, USA). Data are expressed as arithmetic mean ± standard deviation (SD); if other statistical tests were used, the statistical significance per experiment is shown in figure legends.

## Data Availability

The data presented in this study are available on request from the corresponding author. The data are not publicly available due to ethical restrictions.

## Supplementary Materials

The following supporting information can be downloaded at: https://www.mdpi.com/article/10.3390/ijms26157213/s1.
