# Supplementary material for: In-Depth Characterization of L1CAM+ Extracellular Vesicles as Potential Biomarkers for Anti-CD20 Therapy Response in Relapsing–Remitting Multiple Sclerosis
Source: Int J Mol Sci. 2025 Jul 25;26(15):7213. doi: 10.3390/ijms26157213 (PMC12347089; doi:10.3390/ijms26157213)
Supplement: Supplementary file 1 [file ijms-26-07213-s001.zip › ijms-3741148-supplementary.pdf]

Supplementary information

In-depth characterization of L1CAM$^+$ blood extracellular vesicles as potential biomarkers for anti-CD20 therapy response in relapsing–remitting multiple sclerosis

Shamundeeswari Anandan [1,2]*, Karina Maciak [3], Regina Breinbauer [4], Laura Otero [5], Giancarlo Feliciello [6], Nataša Stojanović Gužvić [6], Oivind Torkildsen [1,2], Kjell-Morten Myhr [1,2]

[1] Department of Clinical Medicine, University of Bergen, Bergen, Norway

[2] Neuro-SysMed, Department of Neurology, Haukeland University Hospital, Bergen, Norway

[3] University of Lodz, Faculty of Biology and Environmental Protection, Department of General Biochemistry, Poland

[4] Faculty of Medicine, Friedrich-Alexander-University Erlangen-Nuremberg (FAU), Erlangen, Germany

[5] Neurology and Cerebrovascular Diseases Group, Neurology Department, Neurosciences Area, La Paz Hospital Institute for Health Research, Madrid, Spain

[6] Fraunhofer Institute for Toxicology and Experimental Medicine (ITEM-R), Personalized Tumor Therapy, Regensburg, Germany

* Correspondence: Shamundeeswari.Anandan@uib.no; samanandhan@gmail.com

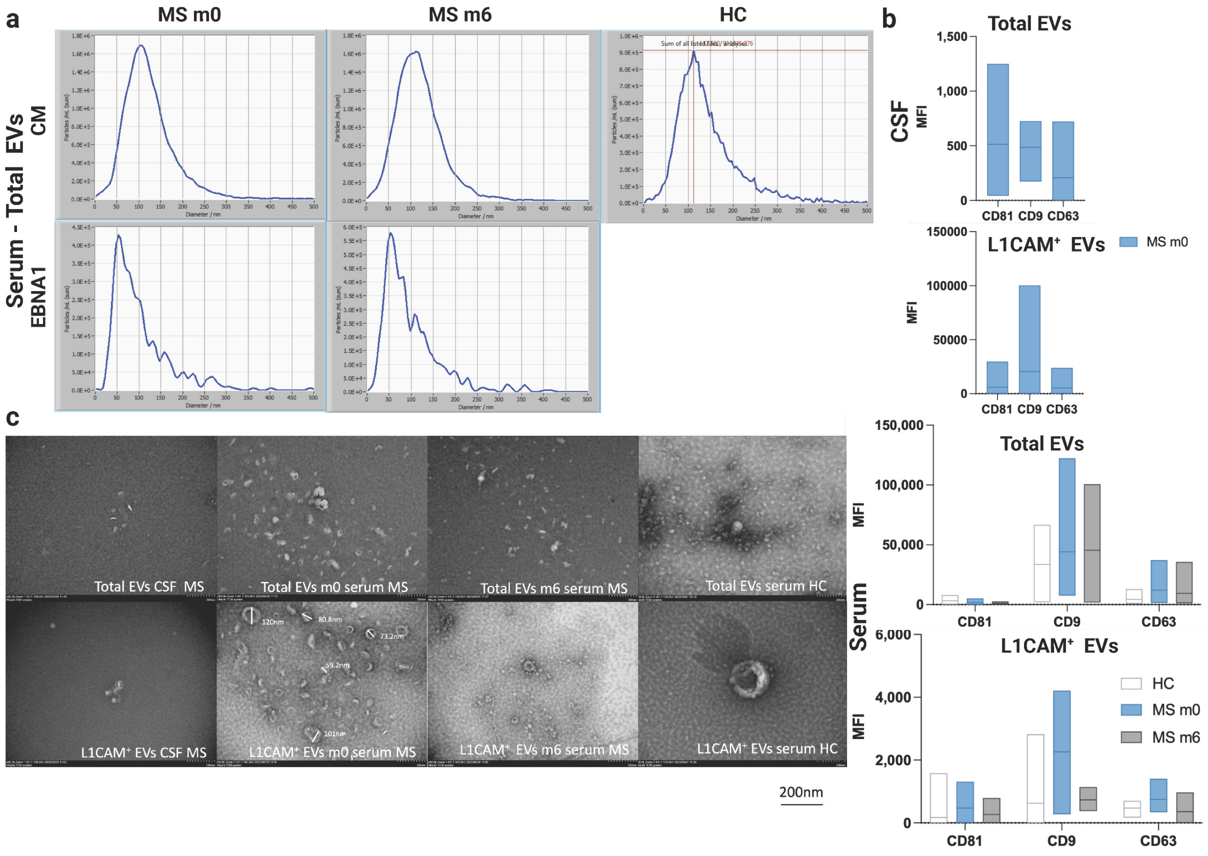

Supplementary Figure S1. Characteristics of serum and CSF EVs (pilot experiments; *n=5*): (a) NTA analysis—total EV concentration and EBNA1^{+} EV concentration in serum total EVs and (b) tetraspanin (CD81, CD9, and CD63) (bead population numbers: 65, 53, and 56 respectively—flow cytometry analysis) expression profile of MS patients (month 0—m0 and month 6—m6) and healthy controls, with total EVs and L1CAM^{+} EVs expressed in mean fluorescence intensities (MFIs), as floating bar plots with min. and max. ranges.

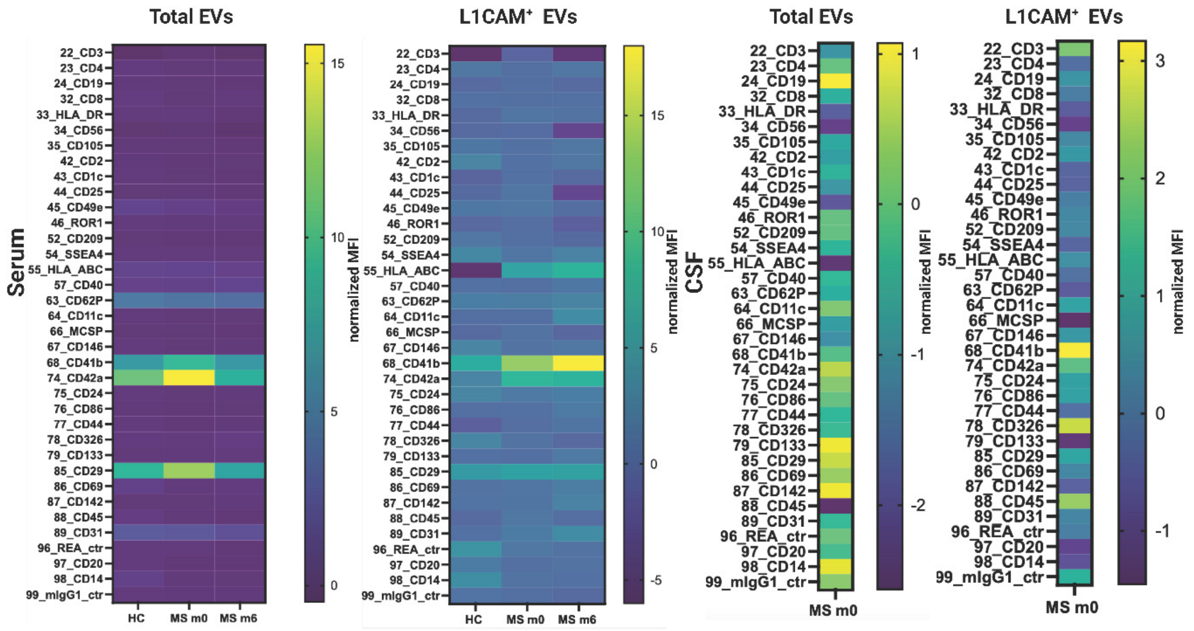

Supplementary Figure S2. Pilot experiments (*n=5*): Surface immune profiling of total and L1CAM$^+$ EVs shown as heatmaps (EV markers' geometric mean fluorescence intensity (MFI) was normalized to the mean MFI for specific EV markers (CD9, CD63, and CD81), obtaining normalized MFI values) from CSF and serum samples comparing MS patients (m0. m6) and healthy controls.

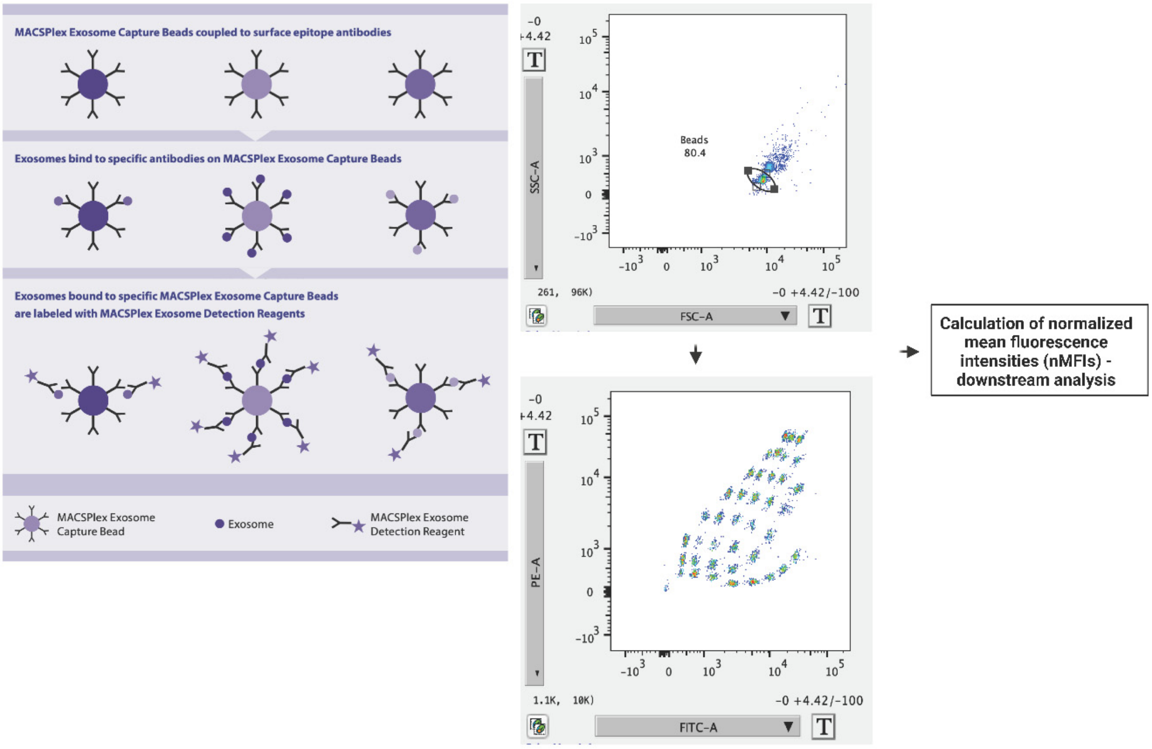

Supplementary Figure S3. General principle and data analysis procedure (MACSPlex Exosome Kit—flow cytometry analysis)—initial gating strategy for the detection of MACSPlex Exosome Capture Bead populations (Supplementary Table 6). Following initial gating strategy, all downstream analyses were based on normalized geometric mean fluorescence intensity (normalized MFI) values. In short, a blank control composed of only MACSPlex buffer, incubated with beads and MACSPlex exosome detection reagents (CD9, CD63, and CD81), was used to measure the background signal. Each EV marker's geometric mean fluorescence intensity (MFI) was normalized to the mean MFI for specific EV markers (CD9, CD63, and CD81), obtaining normalized MFIs.

Supplementary Table S1: Overview of proximity extension assay (PEA) flex panel (21 proteins).

Protein name (gene name)	UniProt No
Transforming growth factor beta-1 proprotein (TGFB1)	P01137
Tumor necrosis factor (TNF)	P01375
Interferon gamma (IFNG)	P01579
C-X-C motif chemokine 10 (CXCL10)	P02778
Interleukin-6 (IL6)	P05231
Granzyme B (GZMB)	P10144
Interleukin-8 (CXCL8)	P10145
C-C motif chemokine 3 (CCL3)	P10147
T-cell-specific surface glycoprotein CD28 (CD28)	P10747
C-C motif chemokine 2 (CCL2)	P13500
Interleukin-10 (IL10)	P22301
B-cell antigen receptor complex-associated protein beta chain (CD79B)	P40259
Interleukin-17A (IL17A)	Q16552
Triggering receptor expressed on myeloid cells 2 (TREM2)	Q9NZC2
Interleukin-4 (IL4)	P05112
Tumor necrosis factor receptor superfamily member 13B (TNFRSF13B)	O14836
Lysosome-associated membrane glycoprotein 3 (LAMP3)	Q9UQV4
Calbindin (CALB1)	P05937
Neurotrophin-3 (NTF3)	P20783
Glial cell line-derived neurotrophic factor (GDNF)	P39905
Visinin-like protein 1 (VSNL1)	P62760

Supplementary Table S2: Differential expression of proteins in serum L1CAM$^+$ EVs (PEA analysis).

**Protein**	**term**	**est.log2**	**p.value**	**p.value.adj.fdr**
GDNF	HCvsM0	-0.0941196	0.23268804	0.678346865
GDNF	M0vsM6	-0.0147704	0.7526507	0.939593942
IFNG	HCvsM0	0.02144309	0.75380304	0.994986221
IFNG	M0vsM6	0.02475317	0.48272582	0.939593942
**CCL2**	HCvsM0	0.50682571	0.02933236	0.256803366
CCL2	M0vsM6	-0.1466272	0.39091575	0.939593942
TNF	HCvsM0	0.09869598	0.35532455	0.678346865
TNF	M0vsM6	0.1113644	0.17661566	0.939593942
CALB1	HCvsM0	-0.0075376	0.8552049	0.994986221
CALB1	M0vsM6	-0.0058484	0.89434305	0.939593942
IL6	HCvsM0	0.06726025	0.28230612	0.678346865
IL6	M0vsM6	0.02886178	0.42863951	0.939593942

**LAMP3**	HCvsM0	0.13777894	0.03203712	0.256803366
LAMP3	M0vsM6	-0.0582832	0.25771699	0.939593942
TNFRSF13B	HCvsM0	0.05441479	0.65223156	0.994986221
TNFRSF13B	M0vsM6	-0.070706	0.37293476	0.939593942
IL4	HCvsM0	-0.0371312	0.73546297	0.994986221
IL4	M0vsM6	0.0830568	0.3616405	0.939593942
TREM2	HCvsM0	0.01371952	0.9327282	0.994986221
TREM2	M0vsM6	0.0429716	0.69364372	0.939593942
GZMB	HCvsM0	0.15907193	0.29042179	0.678346865
GZMB	M0vsM6	-0.0175532	0.89489016	0.939593942
TGFB1	HCvsM0	-0.1073167	0.17789739	0.678346865
TGFB1	M0vsM6	0.0259024	0.68116695	0.939593942
IL10	HCvsM0	-0.0048669	0.95544899	0.994986221
IL10	M0vsM6	0.0066916	0.93959394	0.939593942
VSNL1	HCvsM0	0.00096556	0.99498622	0.994986221
VSNL1	M0vsM6	0.099796	0.40447806	0.939593942
CCL3	HCvsM0	0.10574815	0.16856023	0.678346865
CCL3	M0vsM6	-0.0313888	0.52947453	0.939593942
CXCL8	HCvsM0	0.0336432	0.81410246	0.994986221
CXCL8	M0vsM6	0.0717256	0.74055353	0.939593942
CXCL10	HCvsM0	-0.1116862	0.31067856	0.678346865
CXCL10	M0vsM6	0.0227836	0.78171444	0.939593942
IL17A	HCvsM0	0.04010414	0.76339122	0.994986221
IL17A	M0vsM6	-0.0101408	0.93360394	0.939593942
NTF3	HCvsM0	-0.111682	0.40707273	0.712377278
NTF3	M0vsM6	0.0527444	0.40991603	0.939593942
CD28	HCvsM0	0.1073404	0.33766679	0.678346865
CD28	M0vsM6	0.0450104	0.67133409	0.939593942
**CD79B**	HCvsM0	0.24340203	0.0366862	0.256803366
CD79B	M0vsM6	-0.0273176	0.78811724	0.939593942

Supplementary Table S3: Differential expression of proteins in serum total EVs (PEAanalysis).

**Protein**	**term**	**est.log2**	**p.value**	**p.value.adj.fdr**
GDNF	HCvsM0	-0.0737981	0.45424027	0.55065247
GDNF	M0vsM6	0.0674264	0.25346082	0.59140857
IFNG	HCvsM0	0.26435437	0.23541861	0.5456926
IFNG	M0vsM6	-0.2821888	0.07567835	0.38435591
CCL2	HCvsM0	0.28044315	0.3378097	0.5456926
CCL2	M0vsM6	-0.1588544	0.10810283	0.38435591
TNF	HCvsM0	0.33050819	0.06738182	0.5456926

TNF	M0vsM6	-0.0916212	0.3532373	0.67436212
CALB1	HCvsM0	-0.4075336	0.25467234	0.5456926
CALB1	M0vsM6	0.0010628	0.98180841	0.99376386
IL6	HCvsM0	0.58613001	0.08395289	0.5456926
**IL6**	M0vsM6	-0.3772596	0.03038551	0.3190479
LAMP3	HCvsM0	-0.1333377	0.25570295	0.5456926
LAMP3	M0vsM6	0.1169996	0.14330118	0.42990354
**TNFRSF13B**	HCvsM0	-0.41383	0.03732411	0.5456926
**TNFRSF13B**	M0vsM6	-0.4955192	0.00046214	0.00970504
IL4	HCvsM0	0.12211743	0.32594406	0.5456926
IL4	M0vsM6	-0.08491	0.33601792	0.67436212
TREM2	HCvsM0	0.17862929	0.45437005	0.55065247
TREM2	M0vsM6	-0.1203196	0.16822516	0.44159106
GZMB	HCvsM0	-0.1521611	0.49918702	0.55065247
GZMB	M0vsM6	0.082164	0.57186559	0.80061182
TGFB1	HCvsM0	0.1794297	0.31240653	0.5456926
TGFB1	M0vsM6	0.0604112	0.54545638	0.80061182
IL10	HCvsM0	-0.3168211	0.2615744	0.5456926
IL10	M0vsM6	-0.062948	0.56091222	0.80061182
VSNL1	HCvsM0	0.37644975	0.20355735	0.5456926
VSNL1	M0vsM6	-0.1660452	0.0595543	0.38435591
CCL3	HCvsM0	-0.0603316	0.7828962	0.7828962
CCL3	M0vsM6	-0.0872548	0.44137415	0.77240476
CXCL8	HCvsM0	-0.1446685	0.45968243	0.55065247
CXCL8	M0vsM6	-0.04623	0.6903423	0.83195444
CXCL10	HCvsM0	0.22963381	0.28755122	0.5456926
CXCL10	M0vsM6	0.0078872	0.9541804	0.99376386
IL17A	HCvsM0	0.29800818	0.10895403	0.5456926
IL17A	M0vsM6	-0.21633	0.10981597	0.38435591
NTF3	HCvsM0	-0.2459383	0.45269616	0.55065247
NTF3	M0vsM6	-0.0456296	0.67513991	0.83195444
CD28	HCvsM0	-0.2994642	0.52443092	0.55065247
CD28	M0vsM6	-0.0009476	0.99376386	0.99376386
CD79B	HCvsM0	-0.15161	0.51094789	0.55065247
CD79B	M0vsM6	-0.0451852	0.7131038	0.83195444

Supplementary Table S4: Assessment of protein expression in CSF total EVs at baseline (PEA analysis).

**Protein**	**term**	**mean NPX (est. log2)**
GDNF	M0	0.26
IFNG	M0	0.38
**CCL2**	M0	9.35

TNF	M0	-0.08
CALB1	M0	5.71
IL6	M0	3.15
LAMP3	M0	-0.07
TNFRSF13B	M0	4.60
IL4	M0	1.23
**TREM2**	M0	10.71
GZMB	M0	0.21
TGFB1	M0	1.10
IL10	M0	0.92
VSNL1	M0	0.65
CCL3	M0	0.86
CXCL8	M0	6.47
CXCL10	M0	5.90
IL17A	M0	0.39
NTF3	M0	0.06
CD28	M0	0.24
CD79B	M0	1.97

Supplementary Table S5: Differential expression of proteins in CSF L1CAM^{+} EVs at baseline (PEA analysis).

**Protein**	**term**	**mean NPX (est. log2)**
GDNF	M0	-0.15
IFNG	M0	0.36
CCL2	M0	0.24
TNF	M0	-0.27
CALB1	M0	-1.22
IL6	M0	1.26
LAMP3	M0	0.04
TNFRSF13B	M0	0.15
**IL4**	M0	1.70
**TREM2**	M0	3.26
GZMB	M0	-1.29
TGFB1	M0	0.65
IL10	M0	0.92

VSNL1	M0	-1.82
CCL3	M0	-0.40
CXCL8	M0	0.08
CXCL10	M0	-0.11
IL17A	M0	0.39
NTF3	M0	0.06
CD28	M0	0.39
CD79B	M0	0.19

Supplementary Table S6: Overview of surface marker antibodies used for the MACSPlex Exosome Kit.

No.	Antibody	Isotype
22	CD3	mIgG2a
23	CD4	mIgG2a
24	CD19	mIgG1
32	CD8	mIgG2a
33	HLA-DRDPDQ	recombinant human IgG1
34	CD56	recombinant human IgG1
35	CD105	recombinant human IgG1
42	CD2	mIgG2b
43	CD1c	mIgG2a
44	CD25	mIgG1
45	CD49e	recombinant human IgG1
46	ROR1	mIgG1κ
52	CD209	mIgG1
53	CD9	mIgG1
54	SSEA-4	recombinant human IgG1
55	HLA-ABC	recombinant human IgG1
56	CD63	mIgG1κ
57	CD40	mIgG1κ
63	CD62P	recombinant human IgG1
64	CD11c	mIgG2b
65	CD81	recombinant human IgG1
66	MCSP	mIgG1
67	CD146	mIgG1
68	CD41b	recombinant human IgG1
74	CD42a	recombinant human IgG1
75	CD24	mIgG1
76	CD86	mIgG1
77	CD44	mIgG1

78	CD326	mIgG1
79	CD133/1	mIgG1κ
85	CD29	mIgG1κ
86	CD69	mIgG1κ
87	CD142	mIgG1κ
88	CD45	mIgG2a
89	CD31	mIgG1
96	REA control	recombinant human IgG1
97	CD20	mIgG1
98	CD14	mIgG2a
99	mIgG1 control	mIgG1
